# Association between i.v. thrombolysis volume and door-to-needle times in acute ischemic stroke

**DOI:** 10.1007/s00415-016-8076-5

**Published:** 2016-03-05

**Authors:** Adrien E. Groot, Ivo N. van Schaik, Marieke C. Visser, Paul J. Nederkoorn, Martien Limburg, Majid Aramideh, Frank de Beer, Caspar P. Zwetsloot, Patricia Halkes, Jelle de Kruijk, Nyika D. Kruyt, Willem van der Meulen, Fianne Spaander, Taco van der Ree, Vincent I. H. Kwa, Renske M. Van den Berg-Vos, Yvo B. Roos, Jonathan M. Coutinho

**Affiliations:** Department of Neurology, Academic Medical Center, Meibergdreef 9, 1105 AZ Amsterdam, The Netherlands; Department of Neurology, VU Medical Center, De Boelelaan 1118, 1081 HZ Amsterdam, The Netherlands; Department of Neurology, Flevoziekenhuis, Hospitaalweg 1, 1315 RA Almere, The Netherlands; Department of Neurology, Medical Centre Alkmaar, Wilhelminalaan 12, 1815 JD Alkmaar, The Netherlands; Department of Neurology, Kennemer Gasthuis, Boerhaavelaan 22, 2035 RC Haarlem, The Netherlands; Department of Neurology, Sint Lucas Andreas Ziekenhuis, Jan Tooropstraat 164, 1061 AE Amsterdam, The Netherlands; Department of Neurology, Tergooi Ziekenhuis, Rijksstraat 1, 1261 AN Blaricum, The Netherlands; Department of Neurology, Slotervaart, Louwesweg 6, 1066 EC Amsterdam, The Netherlands; Department of Neurology, Onze Lieve Vrouw Gasthuis, Oosterpark 9, 1091 AC Amsterdam, The Netherlands; Department of Neurology, Rode Kruis Ziekenhuis, Vondellaan 13, 1942 LE Beverwijk, The Netherlands; Department of Neurology, Westfries Gasthuis, Maelsonstraat 3, 1624 NP Hoorn, The Netherlands; Department of Neurology, Waterland, Waterlandlaan 250, 1441 RN Purmerend, The Netherlands; Department of Neurology, Leiden University Medical Center, Albinusdreef 2, 2333 ZA Leiden, The Netherlands

**Keywords:** Stroke, Thrombolytic therapy, Door-to-needle time, Hospitals, High-volume

## Abstract

Centralization of intravenous thrombolysis (IVT) for acute ischemic stroke in high-volume centers is believed to improve the door-to-needle times (DNT), but limited data support this assumption. We examined the association between DNT and IVT volume in a large Dutch province. We identified consecutive patients treated with IVT between January 2009 and 2013. Based on annualized IVT volume, hospitals were categorized as low-volume (≤24), medium-volume (25–49) or high-volume (≥50). In logistic regression analysis, low-volume hospitals were used as reference category. Of 17,332 stroke patients from 11 participating hospitals, 1962 received IVT (11.3 %). We excluded 140 patients because of unknown DNT (*n* = 86) or in-hospital stroke (*n* = 54). There were two low-volume (total 101 patients), five medium-volume (747 patients) and four high-volume hospitals (974 patients). Median DNT was shorter in high-volume hospitals (30 min) than in medium-volume (42 min, *p* < 0.001) and low-volume hospitals (38 min, *p* < 0.001). Patients admitted to high-volume hospitals had a higher chance of DNT < 30 min (adjusted OR 3.13, 95 % CI 1.70–5.75), lower risk of symptomatic intracerebral hemorrhage (adjusted OR 0.39, 95 % CI 0.16–0.92), and a lower mortality risk (adjusted OR 0.45, 95 % CI 0.21–1.01), compared to low-volume centers. There was no difference in DNT between low- and medium-volume hospitals. Onset-to-needle times (ONT) did not differ between the groups. Hospitals in this Dutch province generally achieved short DNTs. Despite this overall good performance, higher IVT volumes were associated with shorter DNTs and lower complication risks. The ONT was not associated with IVT volume.

## Introduction

Since the benefit of intravenous thrombolysis (IVT) for acute ischemic stroke (AIS) is time dependent [1], considerable effort is made to shorten the time from hospital entry to start of treatment, the so-called door-to-needle time (DNT) [2, 3]. The DNT is increasingly used as a performance indicator to evaluate quality of care [4]. It is often assumed that centralization of IVT in designated stroke hospitals results in shorter median DNTs. In 2009, London reorganized its stroke facilities to this extent and Dutch health insurance providers proposed a similar transformation for the Netherlands in 2013. Furthermore, the widespread introduction of mechanical thrombectomy, of which the efficacy has recently been established, will likely become an important factor in the discussion on centralization of acute stroke care, since this therapy is not available in all hospitals [5]. The current rationale of centralization is that increased IVT volumes help to improve hospital logistics, and thus lead to an optimization of the DNT. This view is challenged, however, by the argument that centralization restricts accessibility to emergency care, and may increase the duration of the pre-hospital phase.

Few studies have examined the association between IVT volume and DNT. A phase IV study in Eastern European countries found no relation between IVT volume and median DNT [6]. In contrast, a British study did show that high-volume hospitals achieved significantly shorter DNT and onset-to-needle times (ONT) when compared to medium- and low-volume hospitals [7]. An analysis of the US get-with-the-guidelines database found similar results [8]. In all three studies, however, the overall DNTs were relatively long. In the US and Eastern European study, only 27 and 38 % of patients had a DNT of less than 60 min, respectively. In the British study, the percentage of patients with a DNT < 60 min varied between 30 and 60 % per hospital.

Despite absence of a policy of centralization, hospitals in the Netherlands in general achieve short DNTs. In a recent nationwide survey [9], the reported median DNT varied between 25 and 61 min, implying that all hospitals treat at least 50 % of patients within 1 h of arrival, in accordance with the “Target: Stroke” initiative of the American Heart Association [2]. The question arises whether higher IVT volumes are still associated with a shorter DNT in a region where all hospitals achieve this DNT benchmark, and thus if centralization can be expected to result in further improvement. The effect on the ONT also warrants investigations, as travel times to hospitals in the Netherlands are relatively short compared to less densely populated countries. The aim of our study, therefore, was to examine the association between IVT volume and the DNT and ONT in a well-defined acute care region in the Netherlands.

## Methods

### Study design and patient selection

We performed a cohort study in the province of North-Holland (surface area of 2600 km^2^; 2.75 million inhabitants). Thirteen of the 17 hospitals in the province perform IVT, all of which were invited to participate. We included consecutive patients with AIS treated with IVT between January 2009 and January 2013. Patients were identified through the hospitals’ prospective IVT registries. Hospitals are encouraged to maintain such a database for performance indicators. In case a registry was lacking, we identified patients through the Dutch financial coding system for hospital care. We excluded in-hospital strokes and patients for whom no DNT could be calculated. Stroke mimics treated with IVT were included. We also recorded the total number of patients with AIS that were admitted in the study period. The institutional review board of the Academic Medical Center approved the study and waived the need for written informed consent from individual patients.

### Data collection

Individual patient data were extracted from the medical records. Stroke severity was scored with the NIH Stroke Scale (NIHSS). For patients in whom the NIHSS was not recorded, we constructed the NIHSS from the history and physical admission notes using an algorithm that was previously shown to be reliable [10]. The following outcomes were collected: symptomatic intracerebral hemorrhage (ICH), infections, seizures, decompressive hemicraniectomy, admission to the intensive care, and in-hospital mortality. Symptomatic ICH was defined according to ECASS III definitions. We also scored the frequency of any ICH within 7 days. Each hospital also received a questionnaire regarding the organization of the IVT procedure.

### IVT times

Times of symptom onset, arrival at hospital, initiation of CT, and start of IVT were collected for all patients. With this information, we constructed the following time intervals: DNT, ONT, onset-to-door and door-to-CT time. IVT times were verified against times recorded in the patient records, emergency department records and logged CT times [11]. When the moment of symptom onset was unknown, we used the time the patient was last seen well.

### Statistical analysis

Based on annualized number of IVT procedures, hospitals were categorized as low-volume (≤24 IVT treated patients per year), medium-volume (25–49) or high-volume (≥50), as done previously [7]. This categorization was decided prior to data collection. Intergroup comparisons were analyzed with Mann–Whitney test or Chi square test.

We used multivariate logistic regression analysis to determine if hospital volume was associated with the DNT, ONT and complication rate. We used the following dependent variables: DNT < 30 min, DNT < 45 min, DNT < 60 min, ONT < 120 min, ONT < 150 min, symptomatic ICH and in-hospital mortality. We adjusted for: age, sex, out-of-hours treatment (defined as arrival on weekends or on weekdays before 8 a.m. or after 6 p.m.), NIHSS, blood pressure, atrial fibrillation, diabetes, hypertension and prior stroke. Low-volume hospitals were used as a reference category in the analyses. For the analysis on DNT, we also included the ONT in the model in a sensitivity analysis. Results are given as adjusted odds ratio (OR) with 95 % confidence intervals (CI).

## Results

Of the 13 invited hospitals, 11 agreed to participate (two academic, nine community hospitals). Nine of 11 hospitals kept a prospective registry of consecutive patients treated with IVT, which included the DNT. All participating hospitals provide IVT 24/7 and are pre-notified by the emergency medical services during transport of a patient who is potentially eligible for IVT therapy. Ten of 11 hospitals use a point-of-care device for INR measurement. In 10/11 hospitals the CT scanner is vacated if a code stroke is being transported to the hospital.

In total, 17,332 patients with AIS were admitted to the participating hospitals during the study period. Of these, 1962 (11.3 %) received IVT. We excluded 86 patients for whom it was not possible to calculate a DNT because of missing IVT times, and 54 patients with an in-hospital stroke. Therefore, 1822 patients were included in the analysis (10.5 % of all patients with AIS). The proportion of patients treated with IVT increased from 9.0 % in 2009 to 10.9 % in 2012.

Median DNT for all included patients was 36 min (IQR 25–53) and in 80.5 % the DNT was below 60 min. A DNT < 45 min was achieved in 63.4 % of patients and a DNT < 30 min in 32.1 %. The overall median onset-to-door time (ODT) was 70 min (IQR 46–114) and the median ONT was 115 min (IQR 85–167).

Based on annualized IVT volumes, two hospitals were categorized as low-volume, five as medium-volume and four as high-volume. The total number of patients included per category was 101 (low-volume), 747 (medium-volume) and 974 (high-volume). The baseline characteristics are provided in Table 1.

**Table 1:** Baseline characteristics

	Annualized IVT volume^a^			*p* value Low vs. med	*p *value Low vs. high	*p* value Med vs. high
	0–24 Low	25–49 Medium	≥50 High			
No. of hospitals (no. of patients)	2 (101)	5 (747)	4 (974)	–	–	–
Male sex	55.4 %	55.7 %	53.6 %	0.963	0.722	0.387
Mean age—year (±SD)	68.6 ± 14.1	70.0 ± 14.1	70.0 ± 14.6	0.442	0.358	0.636
Mean systolic blood pressure—mmHg (±SD)	156.4 ± 26.7	155.9 ± 24.6	154.8 ± 24.5	0.819	0.862	0.267
Mean diastolic blood pressure—mmHg (±SD)	85.3 ± 13.9	86.7 ± 15.6	83.5 ± 16.3	0.611	0.223	<0.001
Median NIHSS (IQR)	9 (5–11)	8 (5–13)	7 (4–12)	0.478	0.698	0.027
Risk factors						
Atrial fibrillation	15.1 %	12.2 %	10.8 %	0.433	0.209	0.355
Diabetes	14.0 %	16.6 %	17.0 %	0.515	0.462	0.853
Hypertension	32.3 %	42.4 %	46.0 %	0.062	0.011	0.137
Smoking (former or current)	48.9 %	47.8 %	48.8 %	0.878	0.984	0.723
Prior stroke or TIA	33.3 %	26.4 %	29.3 %	0.157	0.410	0.194
Stroke mimic	1.0 %	1.1 %	0.3 %	0.941	0.284	0.049

^a^Categorization of hospitals is based on the total number of IVT procedures in a particular hospital during the study period (4 years), divided by four. For instance, low-volume hospitals on average performed less than 25 IVT procedures per year (100 in total). Due to annual variation it is possible that in a single year, a low-volume hospital performed more than 25 IVT procedures

A higher proportion of stroke patients were treated with IVT in high-volume hospitals (12.1 %), compared to the medium-volume (11.3 %, *p* = 0.02) and low-volume (3.8 %, *p* < 0.001) hospitals (Table 2). The DNT was significantly shorter in high-volume centers (median DNT 30 min) compared to both medium-volume (median 42 min, *p* < 0.001) and low-volume hospitals (median 38 min, *p* < 0.001). High-volume hospitals more often achieved a DNT < 30 min compared to the other two groups (43.8 vs. 17.4 %, *p* < 0.001, and 26.7 %, *p* = 0.001). The proportion of patients with a DNT < 45 or <60 min was also significantly higher in high-volume hospitals. In the unadjusted analysis, high-volume centers had a shorter ONT than medium-volume (median 115 vs. 120 min, *p* = 0.03), but a longer ONT than low-volume hospitals (115 vs. 103 min, *p* = 0.04). The in-hospital mortality was significantly higher in low-volume hospitals (16.8 %) compared to the other two groups (8.0 and 8.9 %, *p* = 0.004). High-volume hospitals had lower rates of symptomatic ICH (4.3 %) than medium-volume (5.8 %, *p* = 0.17) and low-volume centers (9.9 %, *p* = 0.013).

**Table 2:** Outcomes

	Annualized IVT volume			*p* value Low vs. med	*p* value Low vs. high	*p* value Med vs. high
	0–24 Low	25–49 Medium	≥50 High			
Proportion treated with IVT	3.8 %	11.3 %	12.1 %	<0.001	<0.001	0.02
DNT^a^	38 (29–59)	42 (32–58)	30 (22–45)	0.139	<0.001	<0.001
ONT^a^	103 (69–161)	120 (90–167)	115 (82–170)	0.002	0.035	0.030
ODT^a^	55 (32–99)	65 (45–105)	75 (48–120)	0.008	<0.001	0.001
Door-to-CT^a^	18 (11–28)	20 (13–29)	12 (7–18)	0.314	<0.001	<0.001
DNT < 60 min	74.3 %	76.7 %	84.1 %	0.586	0.012	<0.001
DNT < 45 min	61.4 %	52.6 %	71.9 %	0.097	0.027	<0.001
DNT < 30 min	26.7 %	17.4 %	43.8 %	0.023	0.001	<0.001
Complications						
In-hospital mortality	16.8 %	8.0 %	8.9 %	0.004	0.011	0.504
Any ICH	13.9 %	7.0 %	5.0 %	0.016	<0.001	0.077
Symptomatic ICH	9.9 %	5.8 %	4.3 %	0.108	0.013	0.168
Pneumonia	11.9 %	8.3 %	7.9 %	0.251	0.164	0.680
Urinary tract infection	4.0 %	7.4 %	6.3 %	0.207	0.357	0.362
ICU admission	5.0 %	3.1 %	3.8 %	0.323	0.570	0.420
Hemicraniectomy	1.0 %	2.1 %	2.2 %	0.438	0.431	0.984
Epileptic seizure	3.0 %	1.7 %	3.4 %	0.394	0.824	0.036

*IVT* intravenous thrombolysis, *DNT* door-to-needle time, *ONT* onset-to-needle time, *ODT* onset-to-door time, *ICH* intracerebral hemorrhage, *IQR* interquartile range, *ICU* intensive care unit

^a^Given as median minutes with IQR between parentheses

In the multivariate analysis, patients admitted to a high-volume hospital had a greater chance of receiving IVT within 30 min of arrival than patients admitted to a low-volume hospital (OR 3.13, 95 % CI 1.70–5.75, Table 3). Similar results were found for a DNT < 45 or <60 min. When we added the ODT to the model as co-variable, the results were essentially the same (DNT < 30 min OR 3.01, 95 % 1.63–5.55; DNT < 45 min OR 2.41, 95 % CI 1.45–4.02; DNT < 60 min OR 2.59, 95 % CI 1.49–4.51). Compared to medium-volume centers, patients admitted to a high-volume hospital also had a significantly greater chance of receiving IVT within 30, 45, or 60 min (*p* < 0.001 for each comparison). There were no differences in DNT between low- and medium-volume hospitals. The ONT did not differ between the three groups in the multivariate analysis. Compared to low-volume hospitals, the risk of a symptomatic ICH (OR 0.39, 95 % CI 0.16–0.92) and in-hospital mortality (OR 0.45, 95 % CI 0.21–1.01) were lower in high-volume hospitals. A similar trend was seen in medium-volume centers compared to low-volume.

**Table 3:** Multivariate analyses

		Unadjusted OR (95 % CI)	Adjusted OR (95 % CI)
DNT < 30 min			
Low-volume	27/101 (27 %)	1.0^a^	1.0^a^
Medium-volume	130/747 (17 %)	0.58 (0.36–0.93)	0.83 (0.45–1.55)
High-volume	427/974 (44 %)	2.14 (1.35–3.38)	3.13 (1.70–5.75)
DNT < 45 min			
Low-volume	62/101 (61 %)	1.0^a^	1.0^a^
Medium-volume	393/747 (53 %)	0.70 (0.46–1.07)	1.13 (0.68–1.87)
High-volume	700/947 (72 %)	1.61 (1.05–2.46)	2.58 (1.56–4.26)
DNT < 60 min			
Low-volume	75/101 (74 %)	1.0^a^	1.0^a^
Medium-volume	573/747 (77 %)	1.14 (0.71–1.84)	1.72 (1.01–2.94)
High-volume	819/974 (84 %)	1.83 (1.14–2.95)	2.74 (1.61–4.69)
ONT < 150 min			
Low-volume	71/101 (70 %)	1.0^a^	1.0^a^
Medium-volume	468/700 (67 %)	0.85 (0.54–1.34)	0.92 (0.54–1.58)
High-volume	653/956 (69 %)	0.91 (0.58–1.43)	1.06 (0.62–1.80)
ONT < 120 min			
Low-volume	59/101 (58 %)	1.0^a^	1.0^a^
Medium-volume	348/700 (50 %)	0.70 (0.46–1.07)	0.66 (0.40–1.10)
High-volume	511/956 (54 %)	0.82 (0.54–1.24)	0.81 (0.49–1.35)
Symptomatic ICH			
Low-volume	10/101 (10 %)	1.0^a^	1.0^a^
Medium-volume	43/745 (6 %)	0.56 (0.27–1.15)	0.45 (0.19–1.07)
High-volume	42/973 (4 %)	0.41 (0.20–0.85)	0.39 (0.16–0.92)
In-hospital mortality			
Low-volume	17/101 (17 %)	1.0^a^	1.0^a^
Medium-volume	60/747 (8 %)	0.43 (0.24–0.77)	0.36 (0.16–0.82)
High-volume	87/973 (9 %)	0.49 (0.28–0.85)	0.45 (0.21–1.01)

Unadjusted and adjusted odds ratios (OR) for different cut-offs of the DNT and ONT are given

*CI* confidence interval, *DNT* door-to-needle time, *ONT* onset-to-needle time

^a^Reference category

## Discussion

Our study shows that hospitals in this province of the Netherlands in general achieve short DNTs for IVT in patients with AIS. Approximately 80 % of all patients were treated within 60 min of hospital arrival. In spite of this overall good performance, a higher IVT volume was associated with a shorter DNT. Symptomatic ICH and in-hospital mortality were also more common in hospitals with a low annual IVT volume. We found no association between the ONT and IVT volume.

The difference in median DNT between high-volume hospitals and the other two groups (approximately 10 min) is smaller than previously observed. In a study by Bray et al., high-volume centers were 22 and 28 min faster than medium- and low-volume hospitals, respectively [7]. Recent data from the safe implementation of thrombolysis in stroke (SITS) registry show an even larger difference between high and low-volume hospitals [12]. The most likely explanation for this discrepancy is the overall low DNT by hospitals in our region, which probably reflects the low level of health inequality in the Netherlands. Low-volume hospitals in our study achieved a similar or better median DNT than hospitals with the highest annual volume in other studies [7, 8]. Since the DNT can only be decreased to a certain extent, it is likely that a floor effect influenced our results.

In contrast to other studies, we found no association between IVT volume and ONT. A number of variables might explain this discrepancy. The difference in median DNT was smaller than in other studies, which would make it more difficult to detect a difference in ONT. There may also have been residual confounding variables for which we were unable to adjust. For instance, we had no information on the transportation distance of patients and this may have differed between low- and high-volume hospitals. Guidelines for paramedics state that a patient with a possible stroke and symptoms duration of less than 4.5 h should be transported to the nearest hospital with the possibility to perform IVT. However, in a situation where the transportation distance to two hospitals is more or less the same, ambulance personnel may be inclined to travel to the larger hospital. Consequently, this would lead to longer transportation times for patients admitted to the high-volume centers, thus partly negating the shorter DNT.

It is important to note that there currently is no policy of centralization for stroke patients in the province of North-Holland. As a result, we cannot assume that the IVT times achieved by the high-volume centers accurately reflect the circumstances of a centralized model. If such a model would be implemented, innovations to improve IVT logistics, especially regarding the pre-hospital phase, are likely to be carried out. Such measures have shown to positively influence the DNT and ONT, and the proportion of patients that receive IVT [13–15]. More importantly, the implementation of endovascular reperfusion therapy will influence the future organization of regional acute stroke care [5, 16–19]. Currently, endovascular treatment is provided mostly by high-volume centers and given the level of complexity and the investments required for implementation of this therapy, it is unlikely that this will change in the near future. While a decentralized model is still possible with endovascular treatment (drip-and-ship model), data suggest that the onset-to-reperfusion time is shorter if the process is centralized (mother ship model) [20].

A concern related to the efforts to shorten the DNT is that this could go at the expense of patient safety. Our data do not seem to substantiate this notion. On the contrary, ICH, the most feared complication of IVT, occurred more often in the hospitals with a longer median DNT. The reason for this is not immediately apparent. A delay in treatment is a known risk factor for symptomatic ICH [21], but since there was no difference in ONT, this cannot be the explanation. Other predictors, such as age, blood pressure and stroke severity were adjusted for in the multivariate analysis [21, 22]. We were unable to adjust for serum glucose and early ischemic changes on imaging, and it is possible that these differed between the groups. Finally, although unlikely, we cannot exclude the possibility that the diagnosis of symptomatic ICH was underestimated in medium- and high-volume hospitals due to practice variations regarding repeat imaging in patients with clinical deterioration.

Our study has several limitations. Despite the fact that almost all hospitals recorded the DNT times prospectively, our research question was retrospectively formulated. Fortunately, less than 5 % of patients had to be excluded because of a missing DNT, our primary outcome measure. It would have been helpful, however, if more data on the pre-hospital phase could have been included in the analysis, such as the time that emergency services were first contacted and ambulance transportation times. Considering the association between IVT volume and in-hospital mortality, it would have been interesting to determine if this association persisted at follow-up, but this information was also not available.

In conclusion, hospitals in the Dutch province of North-Holland in general managed to achieve short median DNTs for AIS. Despite this overall good performance, we found that a higher annual IVT volume was associated with a shorter median DNT, a lower symptomatic ICH and a lower mortality rate. The shorter DNT in high-volume hospitals was offset by a longer onset-to-door time, resulting in a similar median ONT in the different hospital categories.
